# Positive effect of inaudible high-frequency components of sounds on glucose tolerance: a quasi-experimental crossover study

**DOI:** 10.1038/s41598-022-23336-0

**Published:** 2022-11-02

**Authors:** Norie Kawai, Manabu Honda, Emi Nishina, Osamu Ueno, Ariko Fukushima, Rikka Ohmura, Nahiko Fujita, Tsutomu Oohashi

**Affiliations:** 1 Department of Research and Development, Foundation for Advancement of International Science, Tsukuba, Japan; 2 Department of Information Medicine, National Center of Neurology and Psychiatry, Kodaira, Japan; 3 Department of Liberal Arts, The Open University of Japan, Chiba, Japan; 4 Center for Liberal Arts and Basic Education, Edogawa University, Nagareyama, Japan; 5 Graduate School of Arts and Sciences, University of Tokyo, Tokyo, Japan

**Keywords:** Neuroscience, Health care

## Abstract

Although stress significantly impacts on various metabolic syndromes, including diabetes mellitus, most stress management techniques are based on psychological and subjective approaches. This study examined how the presence or absence of the inaudible high-frequency component (HFC) of sounds, which activates deep-brain structures, affects glucose tolerance in healthy participants using the oral glucose tolerance test (OGTT). Sounds containing HFC suppressed the increase in glucose levels measured by incremental area under the curve in the OGTT compared with the otherwise same sounds without HFC. The suppression effect of HFC was more prominent in the older age group and the group with high HbA1c. This suggests that sounds with HFC are more effective in improving glucose tolerance in individuals at a higher risk of glucose intolerance.

## Introduction

Stress significantly impacts on the onset and progression of various noncommunicable diseases, including lifestyle-related diseases^1,2^. However, despite the recognition of the importance of stress management, its individualized presentation tends to undermine scientific approaches. Therefore, stress management must be treated as an adjunct to treatment to ensure comprehensive healthcare. While the guidance on material aspects of healthcare is based on objective numerical values, stress management remains a psychological and subjective approach.

The concept of “Information Medicine,” proposed by the authors of this paper as a new approach to psychiatric and neurological disorders, may be effective in addressing this problem^3–5^. The information processing functions of the brain are mediated through chemical reactions, which can be interpreted as an equivalence of matter and information in the brain. Information Medicine is a complementary strategy to conventional medicine, which deals with brain pathophysiology from the material aspect and demystifies the elucidation of brain health and pathophysiological mechanisms. It entails the development of treatment methods from the information processing aspect of the brain^3,5^. Although several forms of Information Medicine have been practiced as nonpharmacological therapies for managing psychiatric and neurological disorders^6^, they largely rely on the experience of medical practitioners and health caregivers. However, the approach is fraught with a lack of sufficient scientific examination compared with material medicine.

In the course of studies to develop reliable Information Medicine, we found that the sound environment of tropical rainforests, where human genes were evolutionarily formed, is rich in inaudible high-frequency components (HFCs) above 20 kHz, whereas artificial urban environmental sounds are completely devoid of such components^3,5,7^. Additionally, we found that sounds rich in HFC enhance the power of the alpha frequency band of spontaneous electroencephalogram^8–10^ and activate deep-brain regions, including the midbrain and thalamus, statistically significantly more than sounds from the same sound source without HFC^8,9^. Furthermore, we revealed that sounds containing HFC have psychological effects that enhance sound comfort^8–10^ and behavioral effects, such as approach behavior^8,11,12^, and reported a series of these phenomena under the name of the hypersonic effect^5,8–10,13,14^. The discovery of the hypersonic effect significantly impacted the field of the audio and media industry, and the development of acoustic equipment and contents covering a wide bandwidth, including outside the human audible range^15^, which is now widely practiced.

Based on this original research, we envisioned a new health and medical strategy (Information Environmental Medicine) for managing various psychiatric and neurological disorders and stress-induced diseases^3,4^. The emerging strategy is premised on the supplementations and enrichment of the urban information environment using advanced media technology. Based on this concept, our previous studies on rodents indicated that mice reared under an information environment with natural environmental sounds have a longer natural life span than those reared in a standard environment without sounds^16^. This previous finding suggests that enrichment of environments with information may positively affect overall health. In addition, the lifespan extension effect of environmental sound was observed to be more pronounced in male individuals exposed to a more stressful environment due to the formation of an ordered relationship between individuals reared in the same cage, suggesting that sound information enrichment may be involved in stress reduction.

The present study examined the effect of HFC in environmental sounds on glucose tolerance in healthy participants. This serves as a tentative step toward examining the possibility that natural environmental sounds that are rich in HFC may be useful in preventing and treating lifestyle-related diseases, which are influenced by stress and significantly impact the onset and transition of pathological conditions.

## Methods

### Participants

The participants comprised 30 healthy Japanese adults (14 females and 16 males), ranging in age from 31 to 69 years, with a mean age of 56.4 ± 9.4 years. Those diagnosed with diabetes mellitus, in addition to those receiving continuous medical treatment for medical or psychiatric/neurological disorders, were excluded.

### Sound materials and presentation systems

We recorded natural environmental sounds in the virgin forests of the Borneo rainforest to use as sound sources. An originally developed, ultra-broadband sound recording system with Direct Stream Digital (DSD) format of 5.6448 MHz sampling was used for recording. The primary components of the sound sources were insect sounds, birdsong, and the rustling of leaves. The sound sources were rich in inaudible HFC above 20 kHz, with complex fluctuation structures in the time domain of a few milliseconds. The frequency of the sound source reached 150 kHz in the time average and 200 kHz in the instantaneous value. We used repeated rainforest natural environmental sounds edited to 20 min.

Three sound conditions were prepared for the experiment: (a) no sound (NS) condition, (b) high-cut sound (HCS) condition, and (c) full-range sound (FRS) condition. In the NS condition, no sound was presented, except for the background noise in the laboratory, such as quiet fan noise. In the HCS condition, the above sound sources were low-pass filtered (a crossover frequency of 20 kHz and a cutoff attenuation of − 200 dB/oct) to remove the inaudible HFC. In contrast, in the FRS condition, the original sound sources were used without any frequency restriction. According to our previous studies, a necessary condition for the emergence of the hypersonic effect stipulates that a frequency component between 40 and 100 kHz must reach the body surface^13,14^. The sound source for the FRS condition used in this study met this condition.

In both the FRS and HCS conditions, sound sources were played back using 5.6448 MHz DSD format on a TASCAM DA3000 (TEAC Corporation, Japan). Four OOHASHI Monitor Op.8 (OOHASHI Monitor Op.8 GAIA HSS-801, Action Research Co., Ltd., Japan) were used for playback in an ultrawideband speaker with a built-in amplifier, and a super tweeter unit that can reproduce HFC exceeding the upper limits of the audible range. Its frequency response was from 20 Hz to 120 kHz at − 10 dB. In addition, four super tweeters (HSST-01P, Action Research Co., Ltd., Japan), with a frequency response from 20 to 200 kHz, were used to enhance the power for the HFC. The frequency spectra of the sounds measured at the participants’ positions are provided as Supplementary Fig. S1 online.

To approximate the natural sound environment of a tropical rainforest, playback speakers and super tweeters were placed in all directions relative to the participants’ positions. In addition, to enhance the comfort of the participants, the sound presentation equipment and cables were made inconspicuous using plants, paintings, and other decorative items depicting the tropical natural environment.

### Glucose measurement

The effect of sound information on glucose tolerance was evaluated using an oral glucose tolerance test (OGTT). The incremental area under the curve (iAUC) of the measured glucose profile during OGTT^17,18^ was employed as the primary endpoint. In a typical OGTT, blood glucose levels are measured by venous blood sampling; however, the psychological stress caused by pain and tension of frequent blood sampling can significantly affect the blood glucose levels. Recently, methods have been developed to easily measure blood glucose levels using sensors attached to the upper arm as diagnostic aids in medical practice. In this study, we used a simple glucose meter [the FreeStyle LibrePro (FSL), Abbott, USA] to examine the glucose levels. A button-shaped waterproof sensor, approximately 3 cm in diameter, with an adhesive and a flexible microneedle attached, was worn on the upper arm, which could automatically and painlessly measure glucose levels every 15 min for up to 14 days continuously.

This device intermittently measures the concentration of glucose in the subcutaneous interstitial fluid [i.e., flush glucose monitoring (FGM)]. Given that glucose moves freely between the capillaries and the interstitial fluid, the glucose concentration in the interstitial fluid correlates with the glucose concentration in the blood and can be used to measure blood glucose levels. Glucose levels measured by FGM correlate well with those measured by authentic blood collection but there is an average error of 14.7% with plasma glucose measurements^19^. In addition, when glucose is orally ingested, the variation in glucose concentration in the interstitial fluid has a time lag of several minutes compared with the variation in glucose concentration in the blood^20^. Since such limitations associated with FGM are expected to equally affect measurements of all sound conditions, we can compare glucose levels among different sound conditions.

### Experimental procedure

All participants were informed about the experiment both orally and in writing, and they provided their written consent. The study was approved by the National Center of Neurology and Psychiatry Ethics Committee (approval number B2021-070), and all experiments were performed in accordance with Declaration of Helsinki. Participants were fitted with a sensor on their upper arm at least one day prior to the commencement of the experiment. HbA1c was measured to confirm that there were no major problems with the glucose tolerance of the participants using A1CNOW + (Polymer Technology Systems, Inc., USA), a simple measuring device that facilitates the collection and measurement of blood by fingertip puncture.

A day before the experiment, the participants were instructed to eat dinner by 8:30 pm, after which they were allowed to fast and drink only water. The recommended amount of sleep was at least 7 h, and the participants were instructed not to be sleep deprived or excessively fatigued on the day of the experiment.

Each participant was involved in one experimental condition per day for a total of 3 days. Since glucose level measured by FSL is considered less accurate within 24 h after insertion and after 10 days^19^, the sensor was fitted on each participant at least 24 h before the first experiment and a schedule of the experiments for each participant was arranged so that the three experimental conditions were completed within 10 days. To control for diurnal variations in several hormones that affect blood glucose levels, OGTTs were performed approximately from 8:30 am to 10:30 am in all experimental conditions. An interval of at least 1 day was allowed between experimental days to allow washout effects. Prior to each experiment, the baseline body temperature, blood pressure, and heart rate measurements were taken for a brief physical condition check. After measuring the baseline glucose levels, the participants consumed 75 g of glucose solution (glucose loading). To control the timing of automatic glucose sampling by FSL, the data recorded over the previous 24 h were read back prior to each OGTT to confirm the actual time every 15 min that the device was measuring, and then each participant was instructed to start the glucose oral intake at the measurement time closest to 8:30 am and complete it within 2 min. In the HCS and FRS conditions, the presentation of natural environmental sounds prepared for each sound condition commenced following the ingestion of the glucose solution and was continued for 2 h. In the NS condition, no natural environmental sounds were presented, and the participants were exposed to the background noise of the laboratory for 2 h. During the 2 h of the OGTT, the participants were allowed to stand up moderately from a seated position in order to avoid boredom or irritation; however, they were prohibited from performing strenuous body movements or exercises. After 2 h, the body temperature, blood pressure, and heart rate measurements were repeated. At the conclusion of each experiment, the participants completed a questionnaire regarding their physical condition during the experiment.

During the OGTT, glucose levels were automatically measured every 15 min using FLS. In addition to the four measurement points at 30, 60, and 120 min after glucose loading, which are the measurement points in the standard OGTT, data at every 15 min were added for a total of nine points (before loading and 15, 30, 45, 60, 75, 90, 105, and 120 min after loading), which were used for analyses.

The NS condition was always performed in the first session to familiarize the participants with the experimental procedure. The order of the FRS and HCS conditions was counterbalanced among all participants in the second and third sessions. Of the 25 participants used in the analysis with valid data, 13 performed HCS as the second session and 12 performed FRS as the second session. Participants were not informed which environmental sound condition was being presented to exclude other extraneous effects following the comparative analyses. However, the possibility of order effects was possible for the NS experimental condition.

### Statistical analysis

All statistical analyses were performed with EZR (Saitama Medical Center, Jichi Medical University, Japan), which is a graphical user interface for R (The R Foundation for Statistical Computing, Austria). More precisely, it is a modified version of R commander designed to add statistical functions frequently used in biostatistics^21^.

First, we examined glucose levels during OGTT using two-way repeated-measure analysis of variance (RM-ANOVA) with two within-subject factors of time (eight time points) and experimental conditions (three conditions). Furthermore, we performed the same analysis on glucose level increments during OGTT. Holm’s correction for multiple comparisons was used for between-condition comparisons. The significance level was set at *P* < 0.05.

Then, the iAUC was determined for each participant at each experimental condition. Subsequently, the normality of the data was tested by the Shapiro–Wilk normality test for each experimental condition. The normality was not rejected in any experimental conditions. Outliers were tested using the Smirnov–Grubbs test, and data from participants containing the detected data were excluded from the analyses.

The differences in iAUC due to the experimental conditions were tested using RM-ANOVA with a within-subject factor of experimental condition with three levels (i.e., NS, FRS, and HCS). Because the sphericity of the data was not rejected in any comparisons, the degrees of freedom were not adjusted. Holm correction for multiple comparisons was used for between-condition comparisons. The level of significance was set at *P* < 0.05.

## Results

A total of 25 participants (12 females and 13 males), with an age range of 42–69 (mean 57.6 ± 7.5), completed this study with valid data. The mean glucose levels of 3 OGTTs at standard measurement points (0, 30, 60, and 120 min) and HbA1c of each participant is provided as Supplementary Table S1.

**Figure 1:**
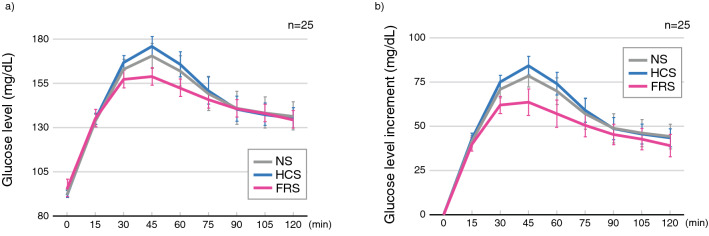
Averaged glucose response curves during the oral glucose tolerance tests under the three experimental conditions. (**a**) mean glucose level across 25 participants (**b**) mean glucose increments from baseline. Error bars indicate standard error of mean. Gray, blue, and pink lines correspond to no sound, high-cut sound, and full-range sound, respectively. A significant main effect of the experimental condition was observed both in the glucose levels and their increments; however no significant interaction was found between time and the experimental condition in either comparison. Between-condition comparisons with Holm’s correction for multiple comparisons showed that the glucose level in the full-range sound condition was significantly lower than that in the high-cut sound condition (*P* = 0.025). Moreover, the glucose level increments in the full-range sound condition were significantly smaller than those in the high-cut sound (*P* = 0.000012) and no sound conditions (*P* = 0.0018).

Figure 1 shows the average and the standard error of mean (S.E.) of the glucose levels (Fig. 1a) and their increments relative to the baseline (Fig. 1b) following oral glucose intake across all participants in three experimental conditions. In both figures, three curves show a similar shape but the degree of increment of glucose levels seems suppressed during the FRS condition compared with the other two conditions. The two-way RM-ANOVA supports this interpretation, revealing a significant main effect of experimental conditions [glucose levels: *F*(2, 384) = 3.59, *P* = 0.029; increments: *F*(2, 384) = 11.6, *P* = 0.000012] and no significant interaction between time and experimental condition [glucose levels: *F*(14, 384) = 0.71, *P* = 0.77; increments: *F*(14, 384) = 0.81, *P* = 0.66]. Between-condition comparisons indicated significantly smaller glucose levels and increments in the FRS condition than in the HCS condition (glucose levels: *P* = 0.025; increments: *P* = 0.000012). The increments were also significantly smaller in the FRS condition than in the NS condition (glucose levels: *P* = 0.16; increments: *P* = 0.0018).

**Figure 2:**
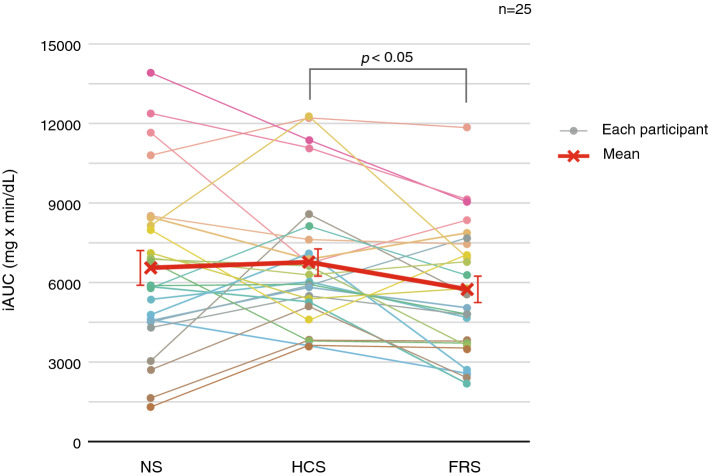
Incremental area under the curve (iAUC) for the three sound conditions for the 25 participants. Red thick line indicates the mean across all participants and error bars indicate the standard error of mean. Colored thin lines indicate each participant’s data. The iAUC was smaller in the full-range sound condition compared with the high-cut sound condition (*P* = 0.039 with Holm correction for multiple comparisons).

Subsequently, the differences in overall responses to oral glucose loading due to experimental conditions were evaluated using iAUC. Figure 2 shows that iAUCs were lower in the FRS condition compared to that found in the HCS condition. RM-ANOVA revealed that the main effects of the three sound conditions showed close to the predefined threshold of statistical significance [*F*(2, 48) = 3.14, *P* = 0.052]. Between-condition comparisons indicated that the iAUC was significantly smaller in the FRS condition than in the HCS condition (HCS vs. FRS: *P* = 0.039; NS vs. FRS: *P* = 0.13; NS vs. HCS: *P* = 0.66).

Although the study excluded patients undergoing treatment for diabetes or those with borderline diabetes and receiving dietary guidance, potential glucose tolerance may vary. The relationship between the variation in potential glucose tolerance and the effect of the sound condition was analyzed. All the 25 participants were divided into two groups based on their age (Table 1, left), with approximately half of the participants in the older group (13 high-age, age range of 59–69 years) and the other half in the younger group (12 low-age, age range of 42–57 years). The mean values and standard deviation of HbA1c in the high-age and low-age groups were 5.55% ± 0.41 and 5.50% ± 0.41, respectively.

**Table 1:** Participant profiles grouped by age and HbA1c.

	Total (valid)	Grouping by age		Grouping by HbA1c	
		High	Low	High	Low
Number	25	13	12	14	11
Gender (female/male)	12/13	7/6	5/7	7/7	5/6
**Age**					
Mean ± SD	57.6 ± 7.5	63.3 ± 3.1	51.3 ± 5.7	60.4 ± 5.1	54.0 ± 8.5
Range	42–69	59–69	42–57	53–69	42–68
**HbA1c**					
Mean ± SD	5.52 ± 0.41	5.55 ± 0.41	5.50 ± 0.41	5.80 ± 0.28	5.17 ± 0.26
Range	4.5–6.5	4.9–6.5	4.5–6.1	5.5–6.5	4.5–5.4

The participants were 30 healthy Japanese adults. Of them, four participants were excluded from the analysis due to the following problems that occurred in at least one of the three measurement conditions: one with sensor detachment, one with noise in the presented sound, one with poor physical condition due to sleeplessness the night before, and one who was found to be regularly taking medication. In addition, one participant was excluded from the analysis because the data were judged as outlier using the Smirnov–Grubbs test. Thus, 25 participants were divided into two groups based on age or value of HbA1c.

**Figure 3:**
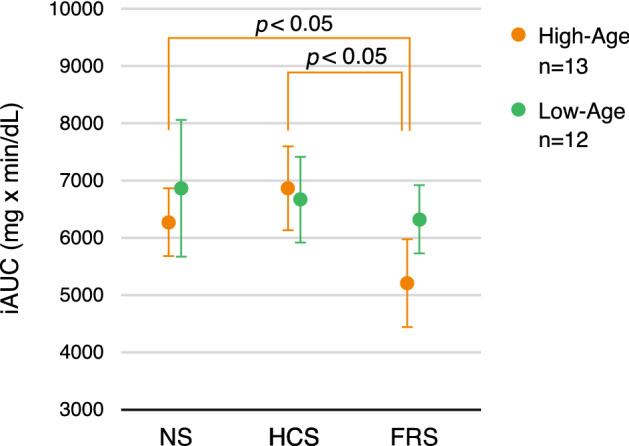
Incremental area under the curve (iAUC) for the three sound conditions for the participants grouped by age. Orange indicates the mean and the standard error of mean of 13 participants in the high-age group and green indicates those of 12 participants in the low-age group. Mean age of high-age and low-age groups was 63.3 (59–69) years and 51.3 (42–57) years, respectively. iAUC was significantly smaller in the full-range sound condition than in both the no sound and high-cut sound conditions in the high-age group, whereas this tendency was not observed in the low-age group.

As shown in Fig. 3, the iAUC was significantly smaller in the FRS condition than in both the NS and HCS conditions in the high-age group. A significant main effect was found [*F*(2, 24) = 5.23, *P* = 0.013], and the FRS condition had a significantly smaller iAUC than that in both the HCS and NS conditions (HCS vs. FRS: *P* = 0.046, NS vs. FRS: *P* = 0.046). There was no significant difference between the NS and HCS conditions (*P* = 0.31). In contrast, these conditional differences were not observed in the low-age group [main effect: *F*(2, 22) = 0.31, *P* = 0.74].

**Figure 4:**
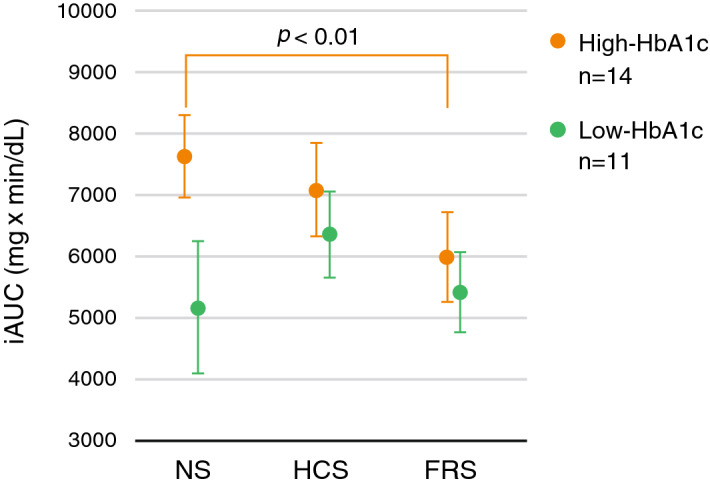
Incremental area under the curve (iAUC) for the three sound conditions for the participants grouped by the value of HbA1c. Orange indicates the mean and the standard error of mean of 14 participants in the high-HbA1c group and green indicates those of 11 participants in the low-HbA1c group. Mean HbA1c of high-HbA1c and low-HbA1c groups was 5.80% (5.5–6.5%) and 5.17% (4.5–5.4%), respectively. iAUC was significantly smaller in the full-range sound condition than in the no sound in the high-HbA1c group, whereas this tendency was not observed in the low-HbA1c group.

In addition, the participants were divided into two groups based on their HbA1c levels (Table 1, right), with 14 in the high-HbA1c group (HbA1c, 5.5 to 6.5%) and 11 in the low-HbA1c group (A1c 4.5 to 5.4%). As shown in Fig. 4, a significant main effect of sound conditions was observed only in the high-HbA1c group [*F*(2, 26) = 5.82, *P* = 0.0081] and iAUC in the FRS conditions showed significant reduction compared with those in the NS condition and a tendency of reduction compared with HCS conditions (NS vs. FRS *P* = 0.0036, HCS vs. FRS *P* = 0.085, NS vs. HCS *P* = 0.35). In contrast, iAUC did not show significant differences [*F*(2, 20) = 1.59, *P* = 0.23] in the low-HbA1c group.

## Discussion

In this study, the FRS condition typically suppressed the increase in glucose levels in the OGTT compared with that in the HCS condition. This tendency was also observed after comparing glucose levels 1 h after glucose loading (Supplementary Fig. S2 online). The suppressive effect of the FRS condition on glucose elevation was more pronounced in the older age group and the group with high HbA1c. However, it was not evident in the younger age group or the group with low HbA1c. Similarly, this tendency was observed when we divided the participants into two groups: high glucose level and low glucose level by OGTT (Supplementary Fig. S3 online). These converging findings imply that sounds with inaudible HFC are more effective in improving glucose tolerance in individuals at a higher risk of glucose intolerance.

### Possible mechanism of effects

It is well experienced in daily practice that stress has a significant impact on glycemic control in patients with diabetes. Many reports have highlighted stress-induced increases in blood glucose levels in patients with type 2 diabetes^22–31^. In addition, a large population-based cohort study of Japanese participants reported a 1.22-fold (women) and 1.36-fold (men) increased risk of developing diabetes in individuals with high subjective stress levels compared with those with low levels^32^. This indicates that stress management influences the pathological transition of patients with diabetes and the prevention of its onset in healthy individuals or potential prediabetics. However, the effects of stress on individuals, both in type and degree, vary so widely^33–35^ that it is practically difficult to study them under experimentally controlled conditions, unlike with pharmacotherapy.

The effects of stress on blood glucose levels are believed to be primarily mediated by neural control from the brainstem and hypothalamus^36,37^. We considered it important to investigate the possibility that acoustic information acting on the hypothalamus and brainstem may have physiological effects on glucose tolerance, independent of psychological effects, rather than primarily reducing subjective stress, which varies considerably among individuals and is difficult to measure objectively.

We have previously reported that deep-brain regions with a close link to the stress response, such as the midbrain, thalamus, and hypothalamus, are activated by the hypersonic effect^5,8,9^. These regions are the highest centers of the autonomic nervous system and endocrine system and are known to affect the immune system. In addition, in a pilot study, we have previously shown that exposure to sounds containing HFC lowers blood adrenaline and cortisol and increases natural killer cell activity compared with exposure to sounds that do not contain such components^5^. Therefore, it is possible that the effect on glucose tolerance observed in this study by sounds rich in inaudible HFC is induced by deep-brain activation^9^, which lowers stress hormones^5^. In the future, it is necessary to further investigate how endocrinological indices and autonomic functions are altered by sounds rich in HFC in relation to blood glucose levels.

We have previously reported that the hypersonic effect does not occur when high-frequency air vibrations are presented to the air-conducting auditory system but only when they are presented to the body surface^14^. Given that the upper limit of the response frequency of Meissner and Patchenyi bodies, which receive vibrations at the body surface, is a few hundred hertz at most^38^, it is not likely that they respond to inaudible high-frequency air vibrations that exceed the upper limit of the audible range. This necessitates an investigation of the molecular mechanism of the acceptance of inaudible high-frequency air vibrations on the body surface from various angles, including the possibility of response from mechanoreceptors in the epidermis. Furthermore, the repair function of skin cells is enhanced when damaged skin cells accept ultrahigh-frequency air vibrations^39^ and that some hormones are produced directly in skin cells by touching the skin^40^. Thus, this study suggests the potential direct effects of the skin responses on the autonomic nervous system and stress hormones.

### Limitations of the interpretation of results

The hypothesis of this experiment was that sound information that activates the deep-brain structure may reduce the stress response and improve glucose tolerance. Therefore, we prioritized the participants’ stress from blood sampling with the understanding that considerable errors would be observed between glucose levels measured using LibrePro and plasma glucose levels measured through blood sampling. In addition, to substitute intermittent blood sampling at regular time intervals in OGTT, we employed FGM in this study rather than continuous glucose monitoring (CGM), which is more accurate than FGM^19,41^ and has been internationally standardized for evaluating glucose tolerance^42^. The increased error associated with FGM would have an equal effect on all sound conditions, leading to difficulties in the detection of any differences between sound conditions harder to detect than when plasma glucose or CGM is used. Despite this disadvantage, the differences in glucose levels due to different sound conditions are significant.

The participants underwent three different sound conditions, but of the three sessions, the NS condition was always conducted first. This was to ensure that the participants were familiarized with the experimental environment and procedures and that their stress was minimized during the experiment. Therefore, we cannot rule out that order effects may have been involved in the observed differences through the comparison of the NS condition with the HCS or FRS conditions. Meanwhile, in the HCS and FRS conditions, the second and third sequences were counterbalanced between participants. Thus, the possibility that order effects were involved in the comparison between the FRS and HCS conditions is extremely low.

Overall, given that this experiment was designed primarily to detect differences between the FRS and HCS conditions, one must practice caution when interpreting the results of the comparison between the NS condition and the other conditions. In contrast, the statistical tests of the data from the current experiment showed significant differences mainly between the FRS and HCS conditions. Simply put, the effect of the difference in sound conditions observed in the present experiment on glucose tolerance can be interpreted as being predominantly due to the presence or absence of an inaudible HFC of sounds.

Moreover, because this experiment did not examine changes in subjective stress levels, a discussion of results in direct relation to subjective stress was not possible. Rather, it is important to interpret these results as a biological response triggered by acoustic information acting on the central nervous system, which is closely associated with stress response, although the participants were not subjectively aware of it. Nevertheless, mixing high-stress traffic noise with inaudible HFCs derived from natural environmental sounds significantly attenuates the discomfort due to the noise^43^. Therefore, it may be plausible to state that the add-on presentation of acoustic information containing inaudible HFCs may also affect subjective stress.

With regard to the relation between age or HbA1c versus the suppression effect, we calculated the correlation between the degree of suppression by HFC (i.e., iAUC of HCS minus iAUC of FRS) and age or HbA1c, but none of them showed a significant correlation. This suggests that the effect of the inaudible HFC on post-OGTT glucose levels is nonlinear when a certain degree of potential risk is present.

### Future direction of the study

As a future direction of this study, we envisage its application to the treatment of type 2 diabetes or borderline diabetes. Educational guidance on lifestyle is extremely important in the treatment of diabetes, prior to the commencement of drug therapy. For diet and exercise therapy, it is possible to indicate targets, etc., by objective numerical values. However, the stress caused by the information environment significantly affects the pathological transition of diabetes via the autonomic nervous system and stress hormones. Nevertheless, although some stress management in patients with diabetes can be evaluated with certain objective numerical values, the majority of them are highly individualized and difficult to approach from a natural scientific perspective because they are caused by complex human relationships and social environments. If the improvement of glucose tolerance by the reception of sounds rich in HFCs can be demonstrated in patients, it could be an effective adjunctive therapy. To demonstrate this, we will conduct a clinical trial of hypersonic effect in people with type 2 or borderline diabetes that are on diet, exercise, and drug therapy, as well as in high-risk healthy individuals such as those with high body mass index, which is a risk factor for type 2 diabetes. It is necessary to verify whether long-term presentation of sounds, including the inaudible HFC, has a clinically ameliorative effect. The safety of this approach, including any negative side-effects, must also be evaluated. Furthermore, it is necessary to develop an acoustic presentation system and highly effective acoustic contents that enable long-term exposure to sounds rich in HFCs in daily life and to conduct field studies outside the laboratory in daily life spaces using such systems. If realized, they are expected to enhance the prospect of information medicine, which approaches diseases by means of information environment enrichment.

## Supplementary Information

Supplementary Information.

## Data Availability

The datasets generated for this study will be provided upon reasonable request to the corresponding authors.
