# Supplementary material for: Positive effect of inaudible high-frequency components of sounds on glucose tolerance: a quasi-experimental crossover study
Source: Sci Rep. 2022 Nov 2;12:18463. doi: 10.1038/s41598-022-23336-0 (PMC9630438; doi:10.1038/s41598-022-23336-0)
Supplement: Supplementary file 1 — Supplementary Information. [file 41598_2022_23336_MOESM1_ESM.pdf]

Supplementary Information for **"Positive effect of inaudible high-frequency components of sounds on glucose tolerance: A quasi-experimental crossover study"**

Norie Kawai*, Manabu Honda*, Emi Nishina, Osamu Ueno, Ariko Fukushima, Rikka Ohmura, Nahiko Fujita, Tsutomu Oohashi

***Co-corresponding author**

Norie Kawai, Ph.D.

Foundation for Advancement of International Science

E-mail: nkawai@fais.or.jp

Manabu Honda, M.D., Ph.D.

Department of Information Medicine,

National Center of Neurology and Psychiatry

E-mail: honda@ncnp.go.jp

Supplementary Figure S1

Frequency spectra of the sounds in this experiment

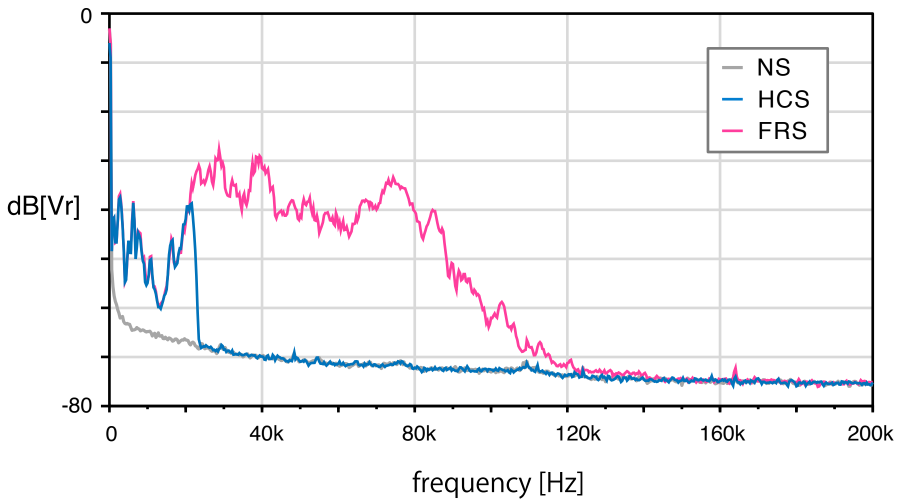

Figure S1.

Frequency spectra of the sounds presented by the sound presentation system used in this experiment. These frequency spectra were obtained by fast Fourier transform analyzer (XN-8100, ONOSOKKI CO., LTD., Japan) of the signal measured with a microphone (Type 4939, Brüel & Kjær, Denmark) placed at the center of the participant's head. The pink and blue lines indicate the power spectra of full-range sound (FRS) and high-cut sound (HCS) conditions, respectively. The gray line indicates the power spectrum of background noise in no sound (NS) condition. FRS contains high-frequency component above 20 kHz, which corresponds to the upper limit of the human audible range, whereas HCS contains only the audible component below approximately 20 kHz. Note that the power spectra below 20 kHz are almost identical between FRS and HCS.

Supplementary Figure S2

Mean glucose level increments
at 1 h and 2 h after glucose loading

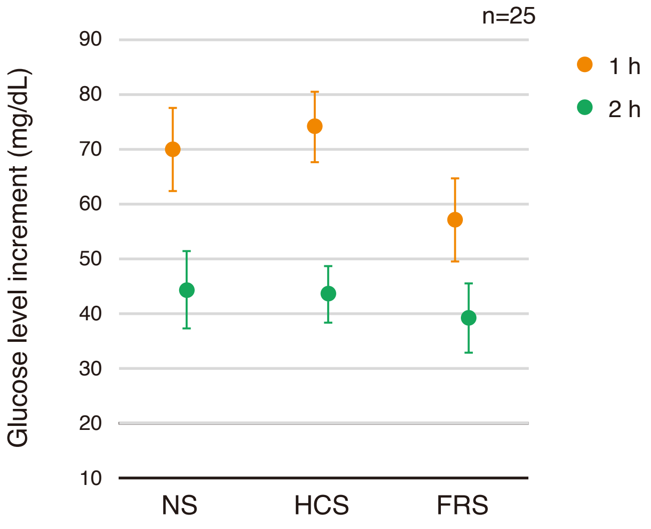

Figure S2.

Mean glucose level increments at 1 h and 2 h after glucose loading for the three sound conditions for 25 participants The orange and green correspond to data of 1 h and 2 h, respectively. Error bars indicate the standard error of mean. At 1 h after glucose loading, RM-ANOVA revealed that the main effects of the three sound conditions showed close to the predefined threshold of statistically significance [$F(2, 48) = 3.18$, $P = 0.051$]. The between-condition comparisons did not show significant difference (HCS vs. FRS: $P = 0.12$; NS vs. FRS: $P = 0.21$; NS vs. HCS: $P = 0.45$). In contrast, at 2 h after glucose loading, no significant difference was observed among experimental conditions [$F(2, 48) = 0.60$, $P = 0.55$].

Supplementary Figure S3

Incremental area under the curve grouped by OGTT values

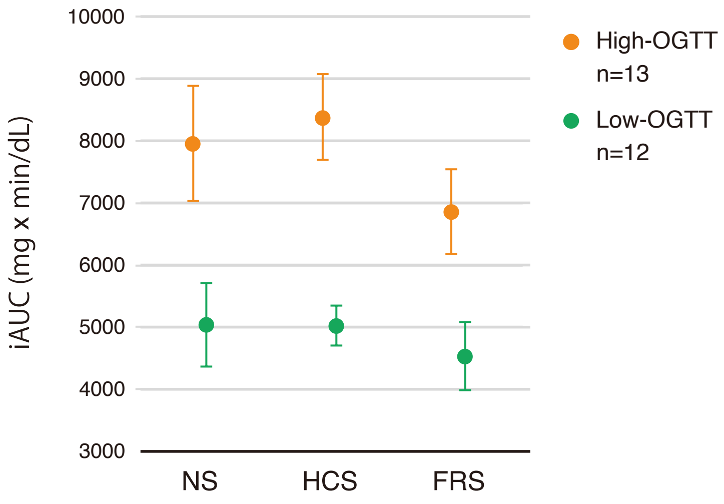

Figure S3.

Incremental area under the curve for the three sound conditions for the participants grouped by the value of oral glucose tolerance test. The green corresponds to the data of 12 participants who were judged as normal-type on all three occasions (low-OGTT group; mean age of 57.8 ± 8.4, 5 female and 7 male) and the orange corresponds to that of 13 participants who were not judged as normal-type on at least one occasion (high-OGTT group; age of 57.3 ± 6.6, 7 female and 6 male). No effect of sound conditions was detected in low-OGTT group [$F(2, 22) = 0.62$, $P = 0.54$], but a nonnegligible difference was detected in high-OGTT group [$F(2, 24) = 2.60$, $P = 0.09$].

Supplementary Table S1

Mean glucose levels across three OGTTs and HbA1c for 25 participants

Participant no.	Mean glucose levels in 3 OGTTs (mg/dL)				HbA1c (%)
	Baseline	30 min	60 min	120 min	
1	83.3	146.3	137.0	123.7	5.9
2	92.3	184.0	196.3	204.3	5.1
3	109.7	178.3	193.0	202.0	6.5
4	87.0	157.0	119.3	113.3	5.6
5	98.7	143.3	166.7	149.7	5.9
6	94.7	184.7	196.0	96.3	5.5
7	97.0	147.3	162.0	131.7	5.5
8	80.3	170.0	176.0	137.7	5.8
9	87.7	179.0	141.7	118.0	5.2
10	96.0	155.7	163.0	119.0	5.5
11	90.3	134.7	143.7	127.0	5.3
12	89.0	146.0	149.0	113.0	5.9
13	79.0	129.0	85.3	117.0	5.4
14	83.7	129.0	109.3	109.7	5.1
15	88.0	163.3	132.0	130.0	5.6
16	101.0	177.0	189.3	109.3	5.4
17	92.7	144.3	129.3	112.0	6.0
18	84.3	185.0	204.0	180.7	5.5
19	87.7	147.3	162.3	139.7	5.4
20	94.7	193.3	180.3	135.3	5.9
21	94.3	173.0	167.3	127.0	4.5
22	100.3	154.7	121.0	113.7	5.4
23	94.7	158.0	145.7	137.0	5.1
24	97.0	161.7	191.7	134.3	4.9
25	121.3	215.3	239.0	200.3	6.1
Grand mean	93.0	162.3	160.0	135.3	5.5
